# (2,3,7,8,12,13,17,18-Octa­ethyl­porphyrinato-κ^4^ *N*)cobalt(II)–2-nitro­benzaldehyde (1/2)

**DOI:** 10.1107/S1600536812036112

**Published:** 2012-08-25

**Authors:** Anissa Mansour, Jean-Claude Daran, Habib Nasri

**Affiliations:** a Département de Chimie, Faculté des Sciences de Monastir, Université de Monastir, Avenue de l’Environnement, 5019 Monastir, Tunisia; b Laboratoire de Chimie de Coordination, CNRS UPR 8241 205 route de Norbonne, 31077 Toulouse, Cedex 04, France

## Abstract

The asymmetric unit of the title compound, [Co(C_36_H_44_N_4_)]·2C_7_H_5_NO_3_, is composed of one half of the complex, arranged about an inversion center, and a complete 2-nitro­benzaldehyde (NBA) mol­ecule. The structure consists of columns that contain inter­leaved mol­ecules of NBA and [Co^II^(OEP)] (OEP is 2,3,7,8,12,13,17,18-octa­ethyl­porphyrin), which are stacked along the *a* axis. The Co^II^ atom is involved in a π inter­action with the ring of the NBA mol­ecule with a centroid–metal distance of 3.508 (6) Å. There is an intra­molecular C—H⋯O hydrogen bond in the NBA mol­ecule.

## Related literature

For the synthesis, see: Scheidt & Tyrk (1994 ▶). For related structures, see: Olmstead *et al.* (2003 ▶); Smirnov *et al.* (1998 ▶); Ben Moussa *et al.* (2011 ▶); Dhifet *et al.* (2010 ▶); Ellison *et al.* (2000 ▶).
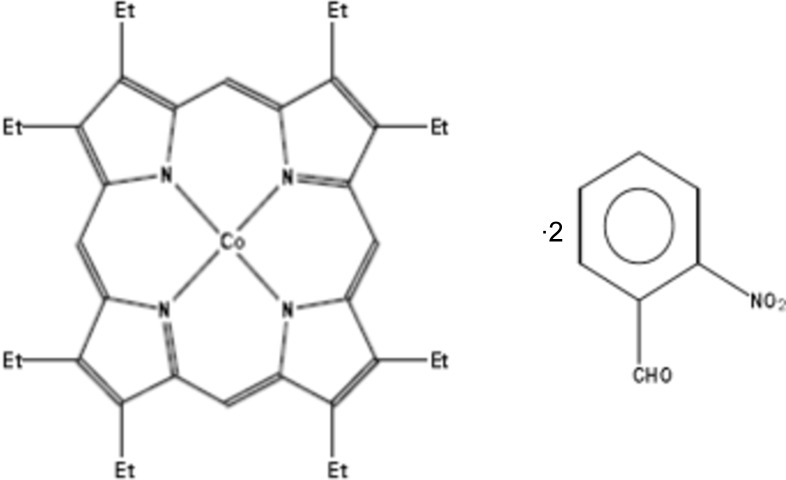


## Experimental

### 

#### Crystal data

[Co(C_36_H_44_N_4_)]·2C_7_H_5_NO_3_
*M* *_r_* = 893.92
Monoclinic, 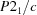

*a* = 10.1952 (11) Å
*b* = 21.2230 (17) Å
*c* = 10.1601 (10) Å
β = 100.771 (9)°
*V* = 2159.6 (4) Å^3^
*Z* = 2
Mo *K*α radiation
μ = 0.46 mm^−1^
*T* = 180 K
0.52 × 0.26 × 0.14 mm

#### Data collection

Agilent Xcalibur diffractometer
Absorption correction: multi-scan (*CrysAlis PRO*; Agilent, 2012 ▶) *T*_min_ = 0.797, *T*_max_ = 0.939
11654 measured reflections
3782 independent reflections
2969 reflections with *I* > 2σ(*I*)
*R*_int_ = 0.038

#### Refinement

*R*[*F* ^2^ > 2σ(*F* ^2^)] = 0.079
*wR*(*F* ^2^) = 0.197
*S* = 1.13
3782 reflections
272 parameters
H atoms treated by a mixture of independent and constrained refinement
Δρ_max_ = 0.99 e Å^−3^
Δρ_min_ = −0.45 e Å^−3^

### 

Data collection: *CrysAlis PRO* (Agilent, 2012 ▶); cell refinement: *CrysAlis PRO*; data reduction: *CrysAlis PRO*; program(s) used to solve structure: *SIR2004* (Burla *et al.*, 2005 ▶); program(s) used to refine structure: *SHELXL97* (Sheldrick, 2008 ▶); molecular graphics: *ORTEPIII* (Burnett & Johnson, 1996 ▶) and *ORTEP-3 for Windows* (Farrugia, 1997 ▶); software used to prepare material for publication: *WinGX* (Farrugia, 1999 ▶) and *SHELXL97*.

## Supplementary Material

Crystal structure: contains datablock(s) I, global. DOI: 10.1107/S1600536812036112/gw2124sup1.cif

Structure factors: contains datablock(s) I. DOI: 10.1107/S1600536812036112/gw2124Isup2.hkl

Additional supplementary materials:  crystallographic information; 3D view; checkCIF report

## Figures and Tables

**Table 1:** Hydrogen-bond geometry (Å, °)

*D*—H⋯*A*	*D*—H	H⋯*A*	*D*⋯*A*	*D*—H⋯*A*
C106—H106⋯O32	1.19 (7)	1.82 (7)	2.701 (7)	126 (5)

**Fig. 1.**
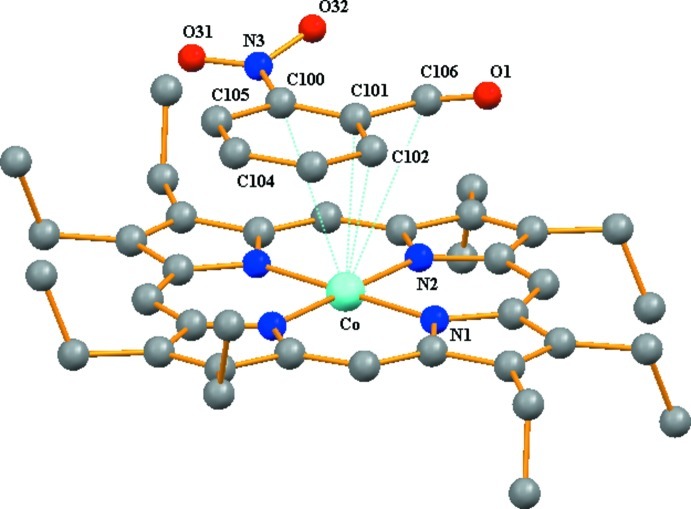
Perspective drawings from the X-ray crystal structure determination of (I) that highlights π-interraction between the [CoII(OEP)] complex and the 2-nitrobenzaldehyde molecule. H atoms have been omitted for clarity.

**Fig. 2.**
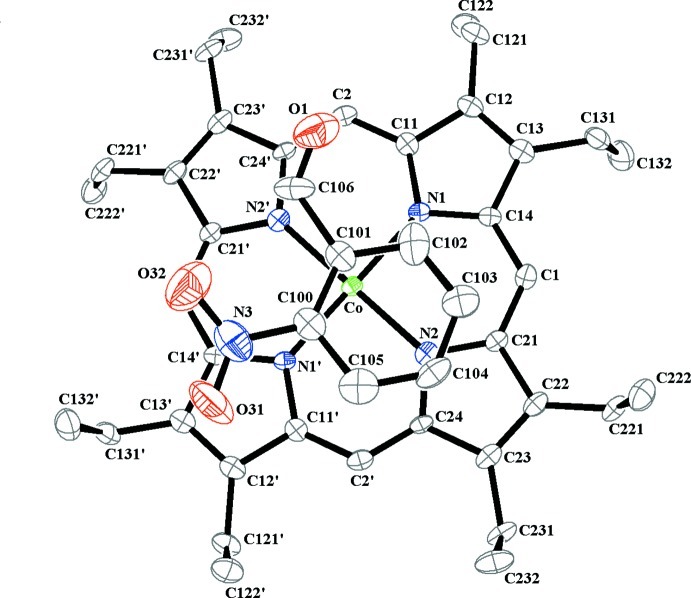
An ORTEP drawing of the structure of [CoII(OEP)].NBA, with the atom-numbering scheme. Dislacement ellipsoids are drawing at 35% and H atoms have been omitted for clarity.Symmetry code: ('): -x, -y, -z.

**Fig. 3.**
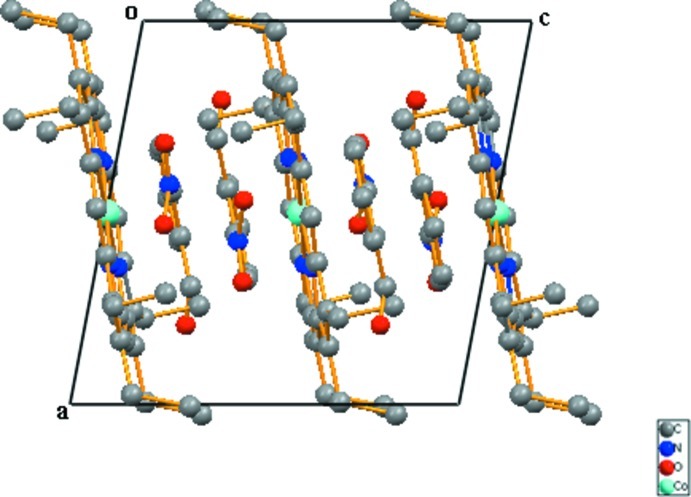
Drawing showing the packing in lattice of [CoII(OEP)].NBA, viewed down the b axis.

## supplementary crystallographic information

### Comment

In continuation of our research on the crystal structures of porphyrin complexes (Ben Moussa *et al.*, 2011; Dhifet *et al.*, 2010) we herein report the synthesis and crystal structure of the title compound. The *x* unit contains one half molecule of [Co^II^(OEP)] and a whole molecule of nitrobenzaldehyde.

For our derivative, the average equatorial cobalt-pyrrole nitrogen atoms distance Co—N_p_ [1.972 (4) Å] is typical for a Co(II) octaethylporphyrin where the porphyrin core is nearly planar (Figure 1) (Scheidt & Tyrk, 1994) and similar to the value of 1.969 (2) Å found in the [Co^II^(OEP)].TNFM (TNFM = (2, 4, 7-trinitrofluoreylidene)malontrile) (Smirnov *et al.*, 1998) and 1.986 (14) Å in the [Co(F_28_TPP)].2tol complex (Tol = toluene and F_28_TPP = tetrakis(pentafluorophenyl)porphyrin) (Olmstead *et al.*, 2003).

It is known that OEP metalloporphyrins can be dimerized as is the case of the [Fe^III^(OEP)(NO)]^+^ complex (Ellison *et al.*, 2000). For this species the distance between two adjacent porphyrinato mean plans is 3.41 Å which indicated a strong π-π interaction. This complex forms a tight cofacial π-π dimer in the solid state.The most interesting feature of (I), is the rather strong π-interaction between the cobalt metal of the [Co^II^(OEP)] and the centroid of the phenyl rings of the nitrobenzaldehyde molecule (Figure 1) where the Co···*Cg* intermolecular distance is 3.508 Å (*Cg* is the centroid of the phenyl ring of the NBA molecule) and the angle between this distance and the perpendicular from the cobalt to the plane of the phenyl is 23.39 ° (Table 2). The cobalt atom is nearly perpendicular to the C101 atom (Figure 2). This structure present a striking resemblance with the one of the [Co^II^(F_28_TPP)].2tol complex (Olmstead *et al.*, 2003) where the cobalt atom is centered roughly at the midpoint at the two adjacent carbons bonds in the toluene rings and the Co—C distances are 3.05 and 3.13 Å. It is noteworthy that the structure of (I) consists of columns that contain interleaved molecules of NBA and [Co^II^(OEP)] which are stacked a long the crystallographic *a* axis (Figure 3).

A unique C—H···O (nitrobenzaldehyde) intramoleculair hydrogen bond of 1.82 (7) Å is found in the structure (Table 1).

### Experimental

[Co^II^(OEP)] (Scheidt & Tyrk, 1994) (100 mg, 0.17 mmol) and nitrobenzaldehyde (190 mg, 1.26 mmol) in 25 ml of chlorobenzene were stirred over night at room temperature. The color changes from red-pink to dark red and crystals of complex (I) were prepared by slow diffusion of hexanes into the chlorobenzene solution.

### Refinement

All H atoms attached to C atoms were fixed geometrically and treated as riding with C—H = 0.96 Å (methyl), 0.97 Å (methylene) or 0.93 Å (aromatic) with *U*_iso_(H) = 1.2*U*_eq_(C_aromatic, methylene_) or *U*_iso_(H) = 1.5*U*_eq_(C_methyl_). The coordinates of the H atom attached to the aldehyde function have been refined freely with *U*_iso_(H) = 1.2*U*_eq_(C).

### Crystal data

**Table:**

[Co(C_36_H_44_N_4_)]·2C_7_H_5_NO_3_	*F*(000) = 942
*M**_r_* = 893.92	*D*_x_ = 1.375 Mg m^−^^3^
Monoclinic, *P*2_1_/*c*	Mo *K*α radiation, λ = 0.71073 Å
Hall symbol: -P 2ybc	Cell parameters from 4505 reflections
*a* = 10.1952 (11) Å	θ = 3.1–28.3°
*b* = 21.2230 (17) Å	µ = 0.46 mm^−^^1^
*c* = 10.1601 (10) Å	*T* = 180 K
β = 100.771 (9)°	Prism, purple
*V* = 2159.6 (4) Å^3^	0.52 × 0.26 × 0.14 mm
*Z* = 2	

### Data collection

**Table:**

Agilent Xcalibur Sapphire1 long-nozzle diffractometer	3782 independent reflections
Radiation source: fine-focus sealed tube	2969 reflections with *I* > 2σ(*I*)
Graphite monochromator	*R*_int_ = 0.038
Detector resolution: 8.2632 pixels mm^-1^	θ_max_ = 25.0°, θ_min_ = 3.2°
ω scans	*h* = −12→12
Absorption correction: multi-scan (*CrysAlis PRO*; Agilent, 2012)	*k* = −25→22
*T*_min_ = 0.797, *T*_max_ = 0.939	*l* = −12→11
11654 measured reflections	

### Refinement

**Table:**

Refinement on *F*^2^	Primary atom site location: structure-invariant direct methods
Least-squares matrix: full	Secondary atom site location: difference Fourier map
*R*[*F*^2^ > 2σ(*F*^2^)] = 0.079	Hydrogen site location: inferred from neighbouring sites
*wR*(*F*^2^) = 0.197	H atoms treated by a mixture of independent and constrained refinement
*S* = 1.13	*w* = 1/[σ^2^(*F*_o_^2^) + (0.0484*P*)^2^ + 9.9571*P*] where *P* = (*F*_o_^2^ + 2*F*_c_^2^)/3
3782 reflections	(Δ/σ)_max_ < 0.001
272 parameters	Δρ_max_ = 0.99 e Å^−^^3^
0 restraints	Δρ_min_ = −0.45 e Å^−^^3^

### Special details

**Table:**

Experimental. Absorption correction: empirical absorption correction using spherical harmonics, implemented in SCALE3 ABSPACK scaling algorithm. CrysAlisPro (Agilent Technologies,2012)
Geometry. All e.s.d.'s (except the e.s.d. in the dihedral angle between two l.s. planes) are estimated using the full covariance matrix. The cell e.s.d.'s are taken into account individually in the estimation of e.s.d.'s in distances, angles and torsion angles; correlations between e.s.d.'s in cell parameters are only used when they are defined by crystal symmetry. An approximate (isotropic) treatment of cell e.s.d.'s is used for estimating e.s.d.'s involving l.s. planes.
Refinement. Refinement of *F*^2^ against ALL reflections. The weighted *R*-factor *wR* and goodness of fit *S* are based on *F*^2^, conventional *R*-factors *R* are based on *F*, with *F* set to zero for negative *F*^2^. The threshold expression of *F*^2^ > 2σ(*F*^2^) is used only for calculating *R*-factors(gt) *etc*. and is not relevant to the choice of reflections for refinement. *R*-factors based on *F*^2^ are statistically about twice as large as those based on *F*, and *R*- factors based on ALL data will be even larger.

### Fractional atomic coordinates and isotropic or equivalent isotropic displacement parameters (Å^2^)

**Table:**

	*x*	*y*	*z*	*U*_iso_*/*U*_eq_	
Co	0.5000	0.0000	1.0000	0.0223 (3)	
N1	0.3667 (4)	0.06811 (18)	0.9652 (4)	0.0219 (8)	
N2	0.6428 (4)	0.06272 (18)	1.0557 (4)	0.0241 (9)	
C1	0.5113 (5)	0.1592 (2)	1.0282 (4)	0.0284 (11)	
H1	0.5142	0.2029	1.0362	0.034*	
C2	0.8358 (5)	−0.0066 (2)	1.1098 (4)	0.0278 (11)	
H2	0.9279	−0.0084	1.1384	0.033*	
C11	0.2321 (5)	0.0628 (2)	0.9185 (4)	0.0249 (10)	
C12	0.1692 (5)	0.1241 (2)	0.9020 (5)	0.0294 (11)	
C13	0.2667 (5)	0.1669 (2)	0.9403 (5)	0.0298 (11)	
C14	0.3887 (5)	0.1319 (2)	0.9805 (4)	0.0235 (10)	
C21	0.6293 (5)	0.1270 (2)	1.0647 (4)	0.0237 (10)	
C22	0.7556 (5)	0.1565 (2)	1.1157 (5)	0.0289 (11)	
C23	0.8479 (5)	0.1099 (2)	1.1354 (5)	0.0294 (11)	
C24	0.7768 (4)	0.0520 (2)	1.0987 (4)	0.0220 (10)	
C121	0.0251 (5)	0.1352 (3)	0.8455 (5)	0.0366 (13)	
H12A	−0.0004	0.1764	0.8734	0.044*	
H12B	−0.0282	0.1039	0.8813	0.044*	
C122	−0.0048 (6)	0.1315 (3)	0.6913 (5)	0.0425 (14)	
H12C	0.0467	0.1628	0.6554	0.064*	
H12D	−0.0981	0.1390	0.6591	0.064*	
H12E	0.0185	0.0905	0.6633	0.064*	
C131	0.2557 (5)	0.2373 (2)	0.9361 (5)	0.0356 (12)	
H13A	0.3161	0.2549	1.0124	0.043*	
H13B	0.1655	0.2494	0.9432	0.043*	
C132	0.2888 (7)	0.2648 (3)	0.8080 (7)	0.0515 (16)	
H13C	0.3767	0.2516	0.7987	0.077*	
H13D	0.2857	0.3099	0.8118	0.077*	
H13E	0.2250	0.2501	0.7325	0.077*	
C221	0.7747 (5)	0.2255 (2)	1.1470 (5)	0.0346 (12)	
H22A	0.7175	0.2497	1.0780	0.041*	
H22B	0.8665	0.2369	1.1451	0.041*	
C222	0.7430 (6)	0.2430 (3)	1.2831 (6)	0.0454 (15)	
H22C	0.6508	0.2341	1.2838	0.068*	
H22D	0.7597	0.2871	1.2994	0.068*	
H22E	0.7984	0.2189	1.3517	0.068*	
C231	0.9941 (5)	0.1149 (3)	1.1924 (5)	0.0351 (12)	
H23A	1.0250	0.1567	1.1744	0.042*	
H23B	1.0422	0.0847	1.1478	0.042*	
C232	1.0261 (3)	0.10290 (19)	1.3431 (2)	0.0475 (15)	
H23C	0.9777	0.1323	1.3878	0.071*	
H23D	1.1201	0.1082	1.3752	0.071*	
H23E	1.0005	0.0607	1.3612	0.071*	
N3	0.4262 (3)	0.10463 (13)	0.6484 (2)	0.0731 (19)	
O1	0.7932 (3)	0.01832 (13)	0.7585 (2)	0.0880 (18)	
O31	0.3191 (3)	0.12778 (13)	0.6159 (2)	0.109 (2)	
O32	0.5322 (3)	0.13821 (13)	0.6534 (2)	0.112 (2)	
C100	0.4442 (2)	0.03814 (12)	0.6568 (2)	0.0412 (14)	
C101	0.5620 (3)	0.00702 (11)	0.7012 (2)	0.0372 (12)	
C102	0.5715 (3)	−0.05699 (11)	0.7001 (2)	0.0581 (19)	
H102	0.6525	−0.0767	0.7326	0.070*	
C103	0.4612 (7)	−0.0916 (3)	0.6509 (6)	0.0528 (16)	
H103	0.4679	−0.1352	0.6466	0.063*	
C104	0.3390 (6)	−0.0629 (3)	0.6070 (5)	0.0442 (15)	
H104	0.2643	−0.0877	0.5766	0.053*	
C105	0.3263 (7)	0.0006 (4)	0.6076 (6)	0.0558 (17)	
H105	0.2441	0.0197	0.5772	0.067*	
C106	0.6934 (6)	0.0416 (4)	0.7524 (6)	0.0517 (17)	
H106	0.683 (7)	0.097 (3)	0.732 (6)	0.062*	

### Atomic displacement parameters (Å^2^)

**Table:**

	*U*^11^	*U*^22^	*U*^33^	*U*^12^	*U*^13^	*U*^23^
Co	0.0218 (5)	0.0259 (5)	0.0186 (4)	−0.0027 (4)	0.0019 (3)	−0.0006 (4)
N1	0.020 (2)	0.029 (2)	0.0165 (18)	−0.0002 (16)	0.0020 (15)	−0.0001 (15)
N2	0.031 (2)	0.030 (2)	0.0122 (18)	−0.0023 (17)	0.0055 (16)	0.0010 (15)
C1	0.036 (3)	0.026 (2)	0.022 (2)	−0.004 (2)	0.002 (2)	0.0014 (19)
C2	0.024 (2)	0.040 (3)	0.019 (2)	−0.004 (2)	0.0026 (18)	0.000 (2)
C11	0.031 (3)	0.030 (3)	0.014 (2)	0.002 (2)	0.0063 (19)	0.0008 (18)
C12	0.031 (3)	0.036 (3)	0.023 (2)	0.008 (2)	0.009 (2)	0.002 (2)
C13	0.033 (3)	0.037 (3)	0.019 (2)	0.005 (2)	0.005 (2)	0.000 (2)
C14	0.022 (3)	0.030 (3)	0.019 (2)	0.0018 (19)	0.0042 (18)	−0.0004 (19)
C21	0.025 (3)	0.029 (3)	0.016 (2)	−0.004 (2)	0.0028 (19)	−0.0007 (18)
C22	0.031 (3)	0.035 (3)	0.021 (2)	−0.012 (2)	0.005 (2)	−0.001 (2)
C23	0.032 (3)	0.039 (3)	0.018 (2)	−0.012 (2)	0.007 (2)	−0.002 (2)
C24	0.018 (2)	0.032 (3)	0.015 (2)	−0.0056 (19)	0.0013 (18)	−0.0001 (18)
C121	0.031 (3)	0.044 (3)	0.035 (3)	0.012 (2)	0.006 (2)	−0.002 (2)
C122	0.039 (3)	0.053 (4)	0.032 (3)	0.007 (3)	−0.003 (2)	0.001 (3)
C131	0.031 (3)	0.036 (3)	0.039 (3)	0.010 (2)	0.005 (2)	−0.003 (2)
C132	0.057 (4)	0.038 (3)	0.061 (4)	0.005 (3)	0.013 (3)	0.011 (3)
C221	0.033 (3)	0.035 (3)	0.035 (3)	−0.017 (2)	0.003 (2)	−0.001 (2)
C222	0.050 (4)	0.042 (3)	0.044 (3)	−0.012 (3)	0.007 (3)	−0.012 (3)
C231	0.026 (3)	0.050 (3)	0.028 (3)	−0.017 (2)	0.002 (2)	−0.003 (2)
C232	0.031 (3)	0.078 (4)	0.029 (3)	−0.008 (3)	−0.005 (2)	−0.001 (3)
N3	0.088 (5)	0.068 (4)	0.067 (4)	0.020 (4)	0.023 (4)	0.003 (3)
O1	0.058 (4)	0.108 (5)	0.094 (4)	−0.016 (3)	0.005 (3)	−0.016 (4)
O31	0.099 (5)	0.085 (4)	0.138 (6)	0.051 (4)	0.005 (4)	0.016 (4)
O32	0.109 (6)	0.104 (5)	0.126 (6)	−0.037 (4)	0.033 (5)	−0.041 (4)
C100	0.052 (4)	0.050 (4)	0.025 (3)	0.008 (3)	0.017 (3)	0.008 (2)
C101	0.047 (3)	0.044 (3)	0.025 (3)	0.008 (3)	0.017 (2)	0.008 (2)
C102	0.088 (6)	0.049 (4)	0.045 (4)	−0.003 (4)	0.033 (4)	0.003 (3)
C103	0.052 (4)	0.069 (4)	0.043 (3)	−0.005 (3)	0.023 (3)	0.005 (3)
C104	0.043 (4)	0.062 (4)	0.029 (3)	−0.023 (3)	0.010 (3)	0.000 (3)
C105	0.060 (4)	0.080 (5)	0.031 (3)	0.001 (4)	0.017 (3)	0.005 (3)
C106	0.028 (3)	0.085 (5)	0.041 (3)	0.006 (3)	0.005 (3)	0.015 (3)

### Geometric parameters (Å, º)

**Table:**

Co—N1	1.970 (4)	C131—H13B	0.9700
Co—N1^i^	1.970 (4)	C132—H13C	0.9600
Co—N2	1.976 (4)	C132—H13D	0.9600
Co—N2^i^	1.976 (4)	C132—H13E	0.9600
N1—C11	1.370 (6)	C221—C222	1.524 (8)
N1—C14	1.376 (6)	C221—H22A	0.9700
N2—C24	1.373 (6)	C221—H22B	0.9700
N2—C21	1.376 (6)	C222—H22C	0.9600
C1—C21	1.373 (7)	C222—H22D	0.9600
C1—C14	1.381 (7)	C222—H22E	0.9600
C1—H1	0.9300	C231—C232	1.526 (5)
C2—C24	1.376 (7)	C231—H23A	0.9700
C2—C11^i^	1.383 (7)	C231—H23B	0.9700
C2—H2	0.9300	C232—H23C	0.9600
C11—C2^i^	1.383 (7)	C232—H23D	0.9600
C11—C12	1.445 (7)	C232—H23E	0.9600
C12—C13	1.350 (7)	N3—O31	1.1858
C12—C121	1.492 (7)	N3—O32	1.2882
C13—C14	1.441 (7)	N3—C100	1.4235
C13—C131	1.499 (7)	O1—C106	1.123 (7)
C21—C22	1.438 (7)	C100—C101	1.3708
C22—C23	1.353 (7)	C100—C105	1.451 (7)
C22—C221	1.503 (7)	C101—C102	1.3620
C23—C24	1.442 (7)	C101—C106	1.531 (7)
C23—C231	1.499 (7)	C102—C103	1.357 (7)
C121—C122	1.542 (7)	C102—H102	0.9300
C121—H12A	0.9700	C103—C104	1.383 (9)
C121—H12B	0.9700	C103—H103	0.9300
C122—H12C	0.9600	C104—C105	1.354 (9)
C122—H12D	0.9600	C104—H104	0.9300
C122—H12E	0.9600	C105—H105	0.9300
C131—C132	1.520 (8)	C106—H106	1.19 (7)
C131—H13A	0.9700		
			
N1—Co—N1^i^	180.000 (1)	C13—C131—H13B	109.1
N1—Co—N2	90.21 (16)	C132—C131—H13B	109.1
N1^i^—Co—N2	89.79 (16)	H13A—C131—H13B	107.9
N1—Co—N2^i^	89.79 (16)	C131—C132—H13C	109.5
N1^i^—Co—N2^i^	90.21 (16)	C131—C132—H13D	109.5
N2—Co—N2^i^	180.00 (16)	H13C—C132—H13D	109.5
C11—N1—C14	104.5 (4)	C131—C132—H13E	109.5
C11—N1—Co	127.9 (3)	H13C—C132—H13E	109.5
C14—N1—Co	127.5 (3)	H13D—C132—H13E	109.5
C24—N2—C21	104.4 (4)	C22—C221—C222	112.9 (4)
C24—N2—Co	127.9 (3)	C22—C221—H22A	109.0
C21—N2—Co	127.6 (3)	C222—C221—H22A	109.0
C21—C1—C14	125.2 (5)	C22—C221—H22B	109.0
C21—C1—H1	117.4	C222—C221—H22B	109.0
C14—C1—H1	117.4	H22A—C221—H22B	107.8
C24—C2—C11^i^	124.6 (4)	C221—C222—H22C	109.5
C24—C2—H2	117.7	C221—C222—H22D	109.5
C11^i^—C2—H2	117.7	H22C—C222—H22D	109.5
N1—C11—C2^i^	124.9 (4)	C221—C222—H22E	109.5
N1—C11—C12	111.2 (4)	H22C—C222—H22E	109.5
C2^i^—C11—C12	123.9 (5)	H22D—C222—H22E	109.5
C13—C12—C11	106.6 (5)	C23—C231—C232	112.7 (4)
C13—C12—C121	128.6 (5)	C23—C231—H23A	109.0
C11—C12—C121	124.8 (5)	C232—C231—H23A	109.0
C12—C13—C14	106.5 (4)	C23—C231—H23B	109.0
C12—C13—C131	128.1 (5)	C232—C231—H23B	109.0
C14—C13—C131	125.3 (5)	H23A—C231—H23B	107.8
N1—C14—C1	124.8 (4)	C231—C232—H23C	109.5
N1—C14—C13	111.2 (4)	C231—C232—H23D	109.5
C1—C14—C13	124.0 (4)	H23C—C232—H23D	109.5
C1—C21—N2	124.6 (4)	C231—C232—H23E	109.5
C1—C21—C22	124.1 (4)	H23C—C232—H23E	109.5
N2—C21—C22	111.2 (4)	H23D—C232—H23E	109.5
C23—C22—C21	106.6 (4)	O31—N3—O32	120.2
C23—C22—C221	128.4 (5)	O31—N3—C100	122.0
C21—C22—C221	124.9 (5)	O32—N3—C100	116.6
C22—C23—C24	106.4 (4)	C101—C100—N3	126.4
C22—C23—C231	128.3 (5)	C101—C100—C105	117.9 (3)
C24—C23—C231	125.2 (5)	N3—C100—C105	115.7 (3)
N2—C24—C2	124.8 (4)	C102—C101—C100	122.5
N2—C24—C23	111.3 (4)	C102—C101—C106	115.0 (3)
C2—C24—C23	123.9 (4)	C100—C101—C106	122.5 (3)
C12—C121—C122	112.1 (4)	C103—C102—C101	119.2 (3)
C12—C121—H12A	109.2	C103—C102—H102	120.4
C122—C121—H12A	109.2	C101—C102—H102	120.4
C12—C121—H12B	109.2	C102—C103—C104	121.0 (6)
C122—C121—H12B	109.2	C102—C103—H103	119.5
H12A—C121—H12B	107.9	C104—C103—H103	119.5
C121—C122—H12C	109.5	C105—C104—C103	121.2 (6)
C121—C122—H12D	109.5	C105—C104—H104	119.4
H12C—C122—H12D	109.5	C103—C104—H104	119.4
C121—C122—H12E	109.5	C104—C105—C100	118.3 (6)
H12C—C122—H12E	109.5	C104—C105—H105	120.9
H12D—C122—H12E	109.5	C100—C105—H105	120.9
C13—C131—C132	112.3 (4)	O1—C106—C101	122.3 (7)
C13—C131—H13A	109.1	O1—C106—H106	120 (3)
C132—C131—H13A	109.1	C101—C106—H106	112 (3)

Symmetry code: (i) −*x*+1, −*y*, −*z*+2.

### Hydrogen-bond geometry (Å, º)

**Table:**

*D*—H···*A*	*D*—H	H···*A*	*D*···*A*	*D*—H···*A*
C106—H106···O32	1.19 (7)	1.82 (7)	2.701 (7)	126 (5)

### Interaction between the Co atom and the phenyl ring of the nitrobenzaldehyde.

Cg1 is the centroid of the C100-C101-C102-C103-C104-C105 phenyl ring. Symmetry codes: (i) 1/2-X, -1/2+Y, 1-Z ; (ii) -1/2+X, 1/2-Y, Z

**Table:**

	Cg···Co (Å)	Co-Perp (Å)	Beta (°)	
Co···Cg1^i^	3.508	-3.219	23.39	
Co···Cg1^ii^	3.508	-3.219	23.39	
